# Analysis and prediction of cardiovascular research hotspots, trends and interdisciplinarity

**DOI:** 10.1136/heartjnl-2025-325877

**Published:** 2025-08-28

**Authors:** Zeye Liu, Ziping Li, Hong Jiang, Guangyu Pan, Wenchao Li, Fengwen Zhang, Wen-Bin Ou-yang, Shouzheng Wang, Cheng Wang, Xuanqi An, Anlin Dai, Ruibing Xia, Yakun Li, Xiaochun Sun, Yi Shi, Chengliang Yin, Xiang-Bin Pan

**Affiliations:** 1 Department of Cardiac Surgery, Peking University People's Hospital, Peking University, Beijing, China; 2 Department of Structural Heart Disease, National Center for Cardiovascular Disease, China & Fuwai Hospital, Chinese Academy of Medical Sciences & Peking Union Medical College, Beijing, China; 3 Zhengzhou University People’s Hospital, Zhengzhou, Henan, China; 4 National Engineering Research Center for Medical Big Data Application Technology, Chinese PLA General Hospital, Beijing, China; 5 University Hospital Munich Department of Cardiology - Medical Clinic and Polyclinic I, Munich, Germany; 6 Academic Medical Centre, Amsterdam, The Netherlands; 7 Medical Innovation Research Department, Chinese PLA General Hospital, Beijing, China

**Keywords:** Cardiovascular Diseases, Education, Medical, Research Design

## Abstract

**Objective:**

Comprehensive data and analyses on cardiovascular research could clarify recent research trends for the academic community and facilitate policy development. We examined publications and reference data to identify research topics, trends and interdisciplinarity for cardiovascular disease (CVD).

**Methods:**

We extracted and clustered text fragments from the titles and abstracts of 2 512 445 publications using artificial intelligence techniques, including natural language processing (NLP) for semantic analysis. Cardiovascular experts identified topics and document clusters based on the output of those semiautomatic methods. We also applied machine learning algorithms to predict the trends over the next 5 years in each field. We examined the crossover between the two cluster groups using citation relationships in the documents.

**Results:**

Research in clinical studies showed the most notable increase; that was followed by research in population and basic studies. The research hotspots were minimally invasive treatments for valve disease, circulatory haemodynamics, and prevention and control of hypertension. The fastest-growing topics were health monitoring, evidence-based medicine and immunotherapy. We found extensive crossover relationships among document clusters for the periods of 2017–2018 and 2020–2021.

**Conclusions:**

This study provides valuable insights into the research hotspots for cardiovascular research, including an increasing emphasis on early disease detection and prevention, exploration of minimally invasive treatments and assessment of risk factors. The research landscape demonstrates signs of interdisciplinarity and integration as reflected in citation relationships. These findings suggest practical implications for optimising resource allocation in healthcare systems, guiding clinical guideline updates and informing policy-making to prioritise high-impact research areas aligned with evolving CVD challenges. Given the evolving global burden of CVD, continuous research and innovation are imperative, with interdisciplinary collaboration assuming a pivotal role in advancing scientific knowledge.

**WHAT IS ALREADY KNOWN ON THIS TOPIC**

- The global burden of cardiovascular diseases continues to rise, driving significant advancements in scientific research.
- Ongoing innovation remains essential to address this challenge.

**WHAT THIS STUDY ADDS**

- Clinical research has shown the most pronounced growth.
- Key emerging trends include a greater emphasis on early disease detection and prevention, the development of minimally invasive treatments and the assessment of risk factors.
- The field also exhibits increasing interdisciplinarity and integration.

**HOW THIS STUDY MIGHT AFFECT RESEARCH, PRACTICE OR POLICY**

- Given the shifting global landscape of cardiovascular disease, sustained research and innovation are critical.
- Promoting interdisciplinary collaboration is essential for advancing scientific discovery and informing clinical and policy decision-making.

## Introduction

 Cardiovascular diseases (CVDs) remain the leading global cause of disease burden.^1 2^ From 1990 to 2019, the number of prevalent CVD cases nearly doubled—from 271 million to 523 million—while deaths rose from 12.1 million to 18.6 million.^2^ This escalating burden underscores the need for intensified research and has shifted public health funding towards scientific innovation and health promotion.^3^

Meta-research, which integrates prior findings and methodologies, is essential for informing policy, guiding education and advancing interdisciplinary knowledge.^3^ However, existing journal classification systems inadequately capture the full scope and evolution of cardiovascular research, making comprehensive reviews challenging. Moreover, the growing role of interdisciplinary work remains underexplored.^3^

To address these limitations, text analysis tools such as CiteSpace and VOSviewer offer classification-independent methods to identify key topics.^4 5^ A 2019 study using natural language processing revealed major research shifts between 2004 and 2013, highlighting the importance of innovation.^4^ Yet, these insights need updating to reflect current trends and the changing disease landscape.

Interdisciplinary collaboration is also increasingly important.^6^ One study of *Nature* publications (1900–2017) found rising cross-disciplinary citations,^6^ but such evaluations remain limited to specific journals, leaving gaps in understanding interdisciplinarity in cardiovascular research.

To fill this gap, we conducted one of the most comprehensive studies to date, analysing over 2.5 million cardiovascular publications from 1944 to 2021—especially the past 40 years. Using text mining, network analysis and clustering, we mapped evolving research themes and their interconnections over time.

## Methods

For detailed method descriptions, please refer to the online supplemental methods. We employed two complementary methods—k-means clustering and latent Dirichlet allocation (LDA)^7 8^—to analyse thematic structures within cardiovascular publications. Text clustering is a foundational step in natural language processing (NLP), aimed at grouping similar documents based on shared lexical patterns. K-means remains a widely used algorithm due to its efficiency, although its performance can suffer in high-dimensional, sparse textual data. To mitigate this, we optimised the number of clusters and employed initialisation and convergence strategies to enhance clustering stability and reproducibility.

To supplement k-means and improve topic coherence, we used LDA, a probabilistic topic modelling method that assigns topic distributions to documents and identifies representative keywords for each topic. This approach improves interpretability and reinforces the semantic structure of clusters. Both methods were implemented using Python libraries, with modelling choices guided by standard evaluation metrics. Our analysis aimed to detect research patterns and map publications into meaningful thematic clusters.

### Data sources

We analysed 2 512 445 cardiovascular publications from 1944 to 2021, including titles, abstracts, keywords and citation data (2011–2021). The dataset was retrieved from the MEDLINE segment of the Web of Science Core Collection using 40 cardiovascular-related MeSH terms (online supplemental table S1), under a data licence from the Chinese Academy of Medical Sciences & Peking Union Medical College.

### Text pre-processing

From an initial pool of 6 468 884 documents, we excluded those without abstracts and removed duplicates using DOI and title, yielding a final dataset of 2 512 445 documents. Titles and abstracts were processed to extract time-related data and noun phrases using a Python-based NLP framework (online supplemental figure S1).

We chose a broad time span (1944–2021) to ensure a sufficient volume of literature and minimise the impact of short-term fluctuations. This period captures key milestones in cardiovascular medicine, such as the introduction of coronary care units (1960), thrombolytic therapy (1970) and percutaneous coronary intervention (1980). Including earlier years helps to contextualise long-term research trends.

No journal-based exclusion criteria were applied to avoid bias from changes in editorial standards, impact factors or research focus over time. Since ‘hot topics’ often span journals regardless of prestige, a comprehensive inclusion approach offers a more accurate reflection of evolving research priorities.

### Modelling and trend prediction

LDA^3 7^ was first applied to all titles and abstracts to uncover topics and group-related documents. Generic terms were filtered to enhance topic specificity. LDA modelling used tokenised keywords and produced 300 topics. Five cardiovascular experts (coauthors of this paper), selected based on domain expertise and publication history, reviewed and refined the topics. Consensus was required to finalise each topic and assign documents to clusters. This process involved expert validation, merging of similar topics and estimation of topic-specific document volumes.

To forecast trends, we used the AutoReg algorithm,^9^ adjusting for missing summary fields in older publications by applying a weighting factor based on the ratio of total to complete documents per year. This enabled estimation of annual topic frequencies and projection of trends across three domains: clinical, basic and population research. We also generated keyword-based word clouds across three time periods (pre-2010, 2010–2020 and post-2020) to identify emerging themes.

Further analysis focused on documents from two time windows: 2017–2018 and 2020–2021. We calculated document similarity using cosine distance and applied a hybrid clustering method.^3 8^ K-means clustering was performed with 20 clusters per period. Five additional cardiovascular experts (also coauthors, but not involved in the LDA step) named each cluster based on its most representative terms. Cluster alignment with LDA topics was visualised using ring diagrams.

To explore niche research areas, we computed yearly inverse document frequency (IDF) for keywords, allowing the identification of underrepresented but emerging topics.

All analyses were conducted in Python V.3.8 on a secure, high-performance computing system.

### Cross-relationship analysis

We analysed citation patterns to assess continuity between document clusters. Specifically, we identified papers from 2017 to 2018 that were cited by those from 2020 to 2021 within each cluster. The results were visualised using a Sankey diagram to illustrate topic evolution and inter-cluster influence.^6^ All glossary of key terms and abbreviations are shown in online supplemental table S4.

## Results

### Research hotspots and evolutionary trends in cardiovascular research

**Figure 1:**
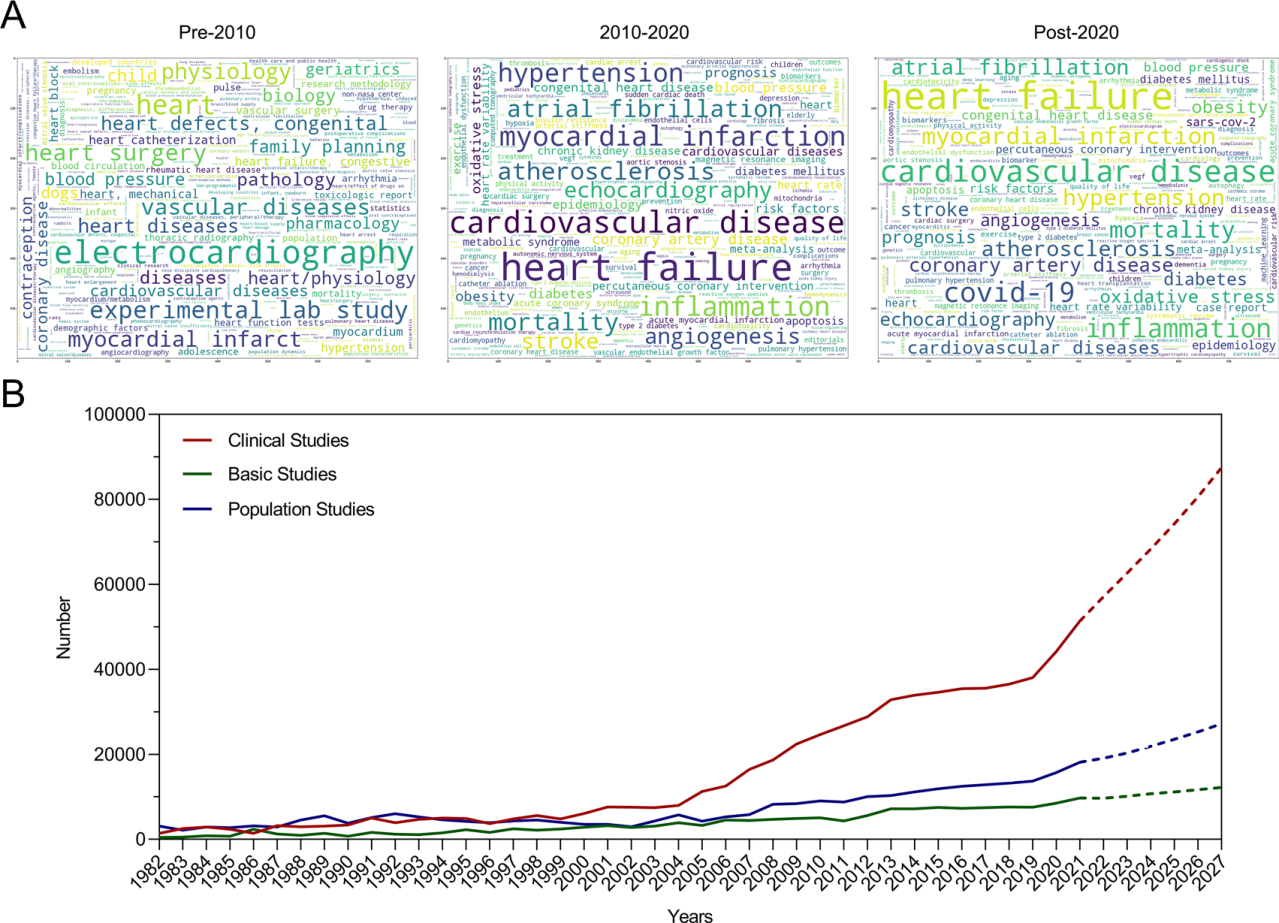
Word clouds representing keywords for three distinct periods and trends in major research categories. **A.** Word clouds representing keywords for three distinct periods (before 2010, 2010–2020, and after 2020): the word size reflects the frequency of appearance. **B.** Temporal trends in the number of studies in three categories (clinical, basic, and population studies).

We generated word clouds representing keywords for three distinct periods: pre-2010, 2010–2020 and post-2020. Figure 1A illustrates the five most frequently occurring keywords in each period.

1. *Pre-2010:* Electrocardiography, experimental lab study, heart surgery, vascular diseases, myocardial infarction.
2. *2010–2020:* Heart failure, cardiovascular disease, myocardial infarction, inflammation and hypertension.
3. *Post-2020:* Heart failure, cardiovascular disease, COVID-19, inflammation, myocardial infarction.

A total of 108 cardiovascular topics were identified, which are listed in online supplemental table S2. These topics were further classified into clinical, basic and population-based studies. For instance, *treatment modalities for coronary artery disease* were categorised as clinical studies, *signalling pathways* were classified as basic studies, and *trends in hypertension prevalence* were regarded as population studies. Notably, since 2004, clinical studies have exhibited the most pronounced growth momentum, whereas basic studies have experienced relatively slower growth, as shown in figure 1B. Given the relatively small number of studies conducted prior to 1982 compared with 2021 and the absence of significant differences in the quantity of studies across various categories during the earlier period, we have only presented the publication data spanning from 1982 to 2021 in figure 1B. Furthermore, we projected the growth trends of these three thematic categories for the next 5 years, with clinical studies projected to continue experiencing the most rapid growth.

**Figure 2:**
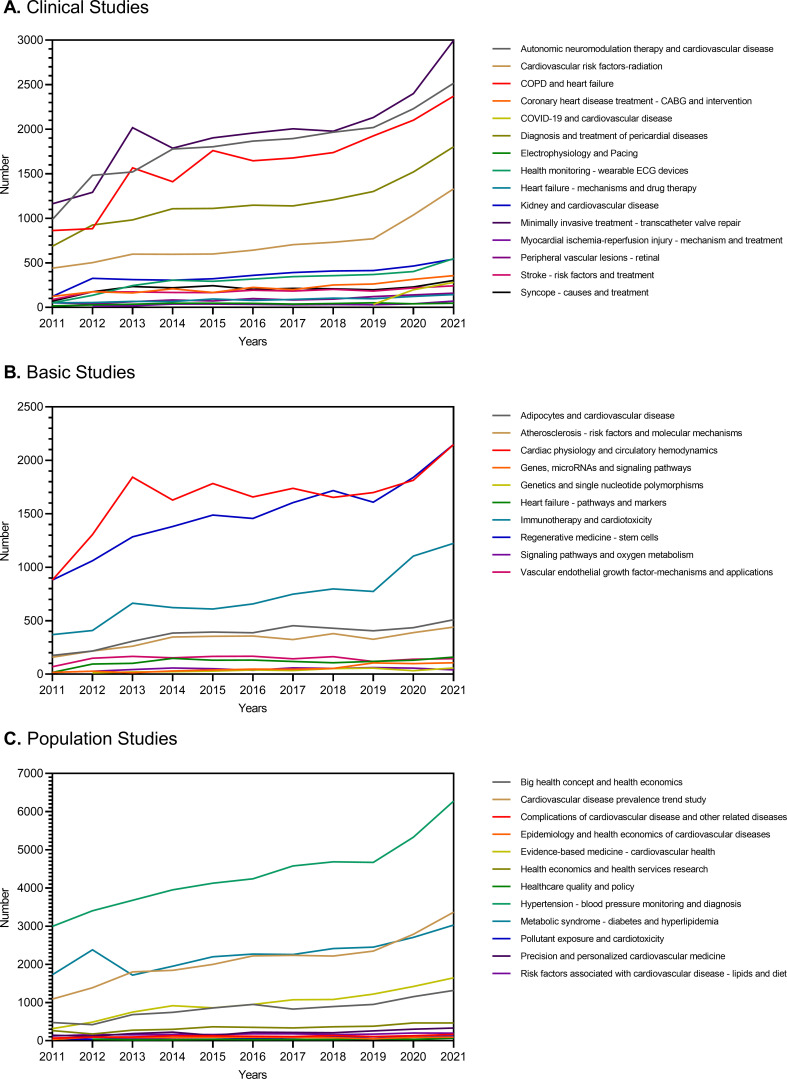
Topics with large growth in 2011–2021.**A.**Topics with significant growth in clinical studies from 2011 to 2021. **B.**Topics with significant growth in basic studies from 2011 to 2021. **C.**Topics with significant growth in population studies from 2011 to 2021.Topics that increased in volume by more than two-fold are shown. Coronary Artery Bypass Grafting (CABG), coronary artery bypass graft; COPD, chronic obstructive pulmonary disease; COVID-19, Coronavirus disease 2019; ECG, electrocardiogram.

Figure 2 highlights the research hotspots that experienced substantial growth in the number of publications between 2011 and 2021. Notably, in clinical studies, *transcatheter valve repair* has emerged as a leading hotspot, likely driven by advancements in technology and device development.^10 11^ Other hot topics include *health monitoring (wearable ECG devices*), *autonomic neuromodulation therapy for CVD*, *chronic obstructive pulmonary disease (COPD), heart failure*, *diagnosis and treatment of pericardial diseases* and *radiation damage (a cardiovascular risk factor)*. It is particularly noteworthy that since 2019, with the onset of the COVID-19 pandemic, *the effects of COVID-19 on CVD* have gained traction as notable research hotspot and are projected to continue growing rapidly until 2027 (figure 2A, online supplemental figure S2A). Basic studies have shown sustained growth in research hotspots such as *cardiac physiology and circulatory haemodynamics*, *regenerative medicine*, *immunotherapy and cardiotoxicity*. Notably, the growth rate of topics on *cardiac physiology and circulatory haemodynamics* was predicted to be slow (figure 2B, online supplemental figure S2B). Within population studies, topics centred around *hypertension* have garnered significant attention, likely due to their high prevalence, low detection rates and potential for late-stage complications.^12^ Topics related to *metabolic syndromes* and *cardiovascular disease prevalence trend studies* also demonstrated notable growth and were predicted to maintain momentum (figure 2C, online supplemental figure S2C). Table 1 presents rapidly growing topics in 2021 compared with those in 2012. It is important to note that the number of publications should be evaluated independently of the growth rate in the number of publications. A full list of 108 LDA topics is presented in online supplemental table S2.

**Table 1:** Major topics in 2012 and 2021

Topics	2012	2021	Percentage change (2012–2021)
	(number of documents)	(number of documents)	
Clinical studies			
Acute coronary syndrome/myocardial infarction—risk factors and diagnosis	3266	4619	41.4%
Acute coronary syndrome—OCT and interventional treatment	939	1413	50.5%
Adjunctive therapy for cardiovascular diseases	449	910	102.7%
Arrhythmia—ventricular arrhythmia diagnosis and treatment	1429	2725	90.7%
Autonomic neuromodulation therapy and cardiovascular disease	1483	2517	69.7%
Cardiovascular risk factors—radiation	503	1332	164.8%
COPD and heart failure	884	2372	168.3%
Coronary artery malformation—diagnosis and treatment	2245	3391	51.1%
Minimally invasive treatment—transcatheter valve repair	1292	2998	132.0%
Pulmonary vascular disease—thromboembolism and pulmonary hypertension	2082	3655	75.6%
Basic studies			
Regenerative medicine—stem cells	1061	2149	102.5%
Immunotherapy and cardiotoxicity	408	1224	200.0%
Cardiac physiology and circulatory haemodynamics	1306	2147	64.4%
Vasoactive drugs—mechanistic studies	802	1004	25.2%
Population studies			
Cardiovascular disease prevalence trend study	1388	3366	142.5%
Big health concept and health economics	421	1316	212.6%
Hypertension—blood pressure monitoring and diagnosis	3402	6276	84.5%
Health economics and health services research	176	464	163.6%
Metabolic syndrome—diabetes and hyperlipidaemia	2381	3028	27.2%

CABG, coronary artery bypass graft; COPD, chronic obstructive pulmonary disease; OCT, optical coherence tomography.

### Large research fields and trends identified through document clustering

**Figure 3:**
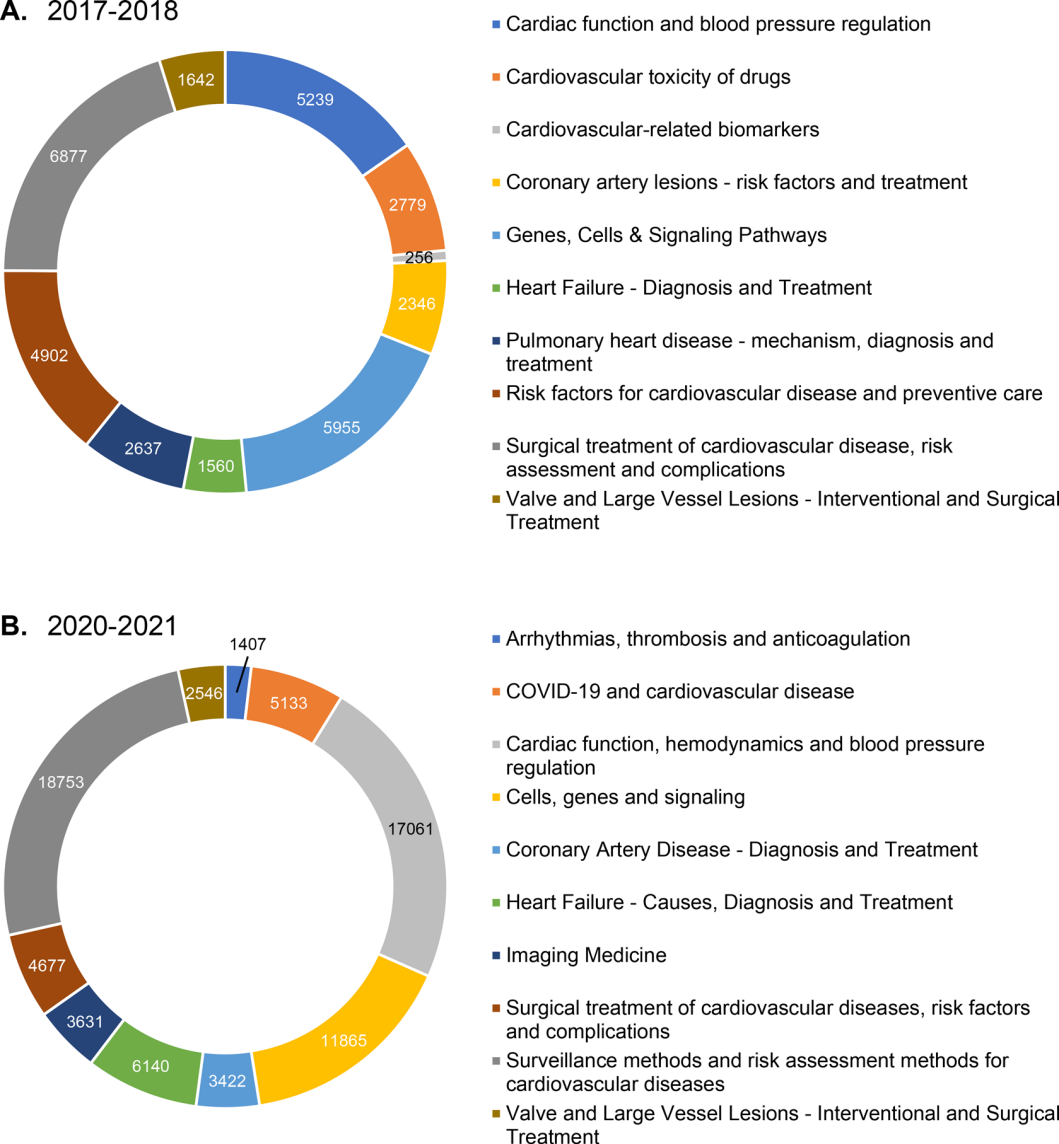
Distribution of document clusters for 2017–2018 and 2020–2021.**A.** In 2017–2018, the ten largest clusters accounted for 95.9% of the total publication output. **B.** In 2020–2021, the ten largest clusters accounted for 96.4% of the total publication output.COVID-19, Coronavirus disease 2019.

Figure 3 illustrates the outcomes of applying a hybrid clustering algorithm to analyse the publication datasets spanning two distinct periods: 2017–2018 and 2020–2021. Ten major clusters were identified within each period. The selection of these two periods was intended to avoid bias, as the COVID-19 pandemic in 2019–2020 had a significant impact on global healthcare systems, with many studies focusing exclusively on COVID-19 and related coronaviruses. Notably, domains such as *cardiac function*, *blood pressure regulation*, *coronary artery disease (lesions*), *surgical treatment of CVD with risk assessment and complications*, and interventional and surgical treatment of valve and large vessel diseases maintained their prominence throughout both periods. Furthermore, basic study topics, including *genes*, *cells* and *signalling pathways*, also constituted a substantial proportion of the two periods. Noteworthy developments in 2020–2021 include the emergence of *COVID-19 and CVD*, and *imaging medicine has* also emerged as a prominent area of research. In contrast, the topic of *cardiovascular toxicity of drugs*, defined in 2017–2018, is no longer identifiable in 2020–2021 (figure 3).

Table 2 presents the most highly associated topics among the ten largest document clusters in each period. Overall, LDA topics provide more detailed insights into the research contained in the clusters and help us understand the changes in topics within the clusters. For instance, the cluster ‘*Valve and large vessel lesions—interventional and surgical treatment’* encompassed critical topics such as the *diagnosis and treatment of vascular malformations* and *aneurysms*, as well as related *surgical and minimally invasive interventional treatment* during 2017–2018. However, from 2020 to 2021, more attention has been paid to the *diagnosis and treatment of congenital cardiovascular malformations*. The cluster ‘*Surgical treatment of cardiovascular disease*’ showed stability across the two periods.

**Table 2:** Cluster names and topics within clusters

2017–2018		2020–2021	
Cluster name	LDA topics	Cluster name	LDA topics
Cardiac function and blood pressure regulation	Vasoactive drugs—mechanistic studies Hypertension—blood pressure monitoring and diagnosis Vascular endothelial cells and angiogenesis Kidney and cardiovascular disease Cardiovascular effects of anaesthesia	Arrhythmias, thrombosis and anticoagulation	Arrhythmia—ventricular arrhythmia diagnosis and treatment Thrombosis and embolism—diagnosis and treatment Electrophysiology and pacing
Cardiovascular-related biomarkers	Biomarkers—diagnosis and prediction Risk factors associated with cardiovascular disease—lipids and diet Heart failure—pathways and markers Metabolism of the cardiovascular system	Cardiac function, haemodynamics and blood pressure regulation	Hypertension—blood pressure monitoring and diagnosis Vasoactive drugs—mechanistic studies Vascular endothelial cells and angiogenesis
Cardiovascular toxicity of drugs	Cardiotoxicity of chemotherapy drugs Immunotherapy and cardiotoxicity Drug-related cardiovascular safety and tolerability Cardiovascular effects of anti-inflammatory drugs	Cells, genes and signalling	Regenerative medicine—stem cells Adipocytes and cardiovascular disease Genes, microRNAs and signalling pathways Signalling pathways and oxygen metabolism Signal transduction and cardiac proteins
Coronary artery lesions—risk factors and treatment	Acute coronary syndrome/myocardial infarction—risk factors and diagnosis Acute coronary syndrome—OCT and interventional treatment Coronary artery malformation—diagnosis and treatment	Coronary artery disease—diagnosis and treatment	Coronary artery malformation—diagnosis and treatment Acute coronary syndrome/myocardial infarction—risk factors and diagnosis Acute coronary syndrome—OCT and interventional treatment Coronary interventional stenting—efficacy and complications
Genes, cells and signalling pathways	Genes, microRNAs and signalling pathways Regenerative medicine—stem cells Adipocytes and cardiovascular disease	COVID-19 and cardiovascular disease	COVID-19 and cardiovascular disease
Heart failure—diagnosis and treatment	Heart failure—mechanisms and drug therapy Heart failure—pathways and markers COPD and heart failure	Heart failure—causes, diagnosis and treatment	COPD and heart failure Heart failure—pathways and markers Heart failure—mechanisms and drug therapy
Risk factors for cardiovascular disease and preventive care	Metabolic syndrome—diabetes and hyperlipidaemia Complications of cardiovascular disease and other related diseases Infectious diseases and the bacterial microenvironment Cardiovascular risk factors—radiation Pollutant exposure and cardiotoxicity Atherosclerosis—risk factors and molecular mechanisms	Imaging medicine	Imaging medicine—clinical applications of echocardiography Imaging medicine—new approaches to cardiovascular ultrasound Imaging medicine—MRI and nuclear medicine Imaging medicine—radiology and CT
Surgical treatment of cardiovascular disease, risk assessment and complications	Valve diseases—surgical treatment and complications Surgical treatment of congenital heart disease and complications Coronary heart disease treatment—CABG and intervention Bleeding and vascular complications	Surgical treatment of cardiovascular diseases, risk factors and complications	Coronary heart disease treatment—CABG and intervention Surgical treatment of congenital heart disease and complications Valve diseases—surgical treatment and complications Bleeding and vascular complications
Valve and large vessel lesions—interventional and surgical treatment	Vascular malformations—diagnosis and treatment Minimally invasive treatment—TAVI Aneurysm—causes and treatment Valve diseases—surgical treatment and complications Minimally invasive treatment—interventional treatment of valve diseases	Surveillance methods and risk assessment methods for cardiovascular diseases	Health monitoring—wearable ECG devices Precision and personalised cardiovascular medicine Cardiac arrhythmias— implantable defibrillator applications and complications
Pulmonary heart disease—mechanism, diagnosis and treatment	COPD and heart failure Pulmonary vascular disease—thromboembolism and pulmonary hypertension	Valve and large vessel lesions—interventional and surgical treatment	Minimally invasive treatment of valve disease Minimally invasive treatment—transcatheter valve repair Vascular malformations—diagnosis and treatment Congenital cardiovascular malformations—diagnosis and treatment Aneurysm—causes and treatment

CABG, coronary artery bypass graft; COPD, chronic obstructive pulmonary disease; LDA, latent Dirichlet allocation; OCT, optical coherence tomography; TAVI, transcatheter aortic valve implantation.

While focusing on the principal research areas, it is imperative not to overlook studies that may have generated relatively few results. Consequently, we computed the IDF for each keyword for each year. This analysis revealed keywords that, while present across multiple years, exhibited a noteworthy spike in frequency during a specific year. Table 3 provides a detailed account of the most frequent keywords in their respective fields for each year within the 2011–2021 period. For example, the most frequent keywords over the last 3 years of this period encompassed *multisystem inflammatory syndrome* (2021), *2019-nCoV* (2020) and *intracranial aneurysm* (2019). Of particular note, *multisystem inflammatory syndrome* is a rare but serious condition associated with COVID-19,^13^ representing the far-reaching impact of the COVID-19 pandemic. A comprehensive list of keywords spanning 1944–2021 is presented in online supplemental table S3.

**Table 3:** The most frequent topics from 2011 to 2021

Year	Research topics
2021	Coronavirus disease 2019
2020	Intracranial aneurysm
2019	Cranial nerve
2018	Reflex syncope
2017	Gerotarget
2016	Angiogenesis effect
2015	Paediatric interventions
2014	Left ventricle/ventricular
2013	Intensity-modulated radiation therapy
2012	Antiarrhythmic therapy
2011	Whole blood viscosity

### Cluster crossover

**Figure 4:**
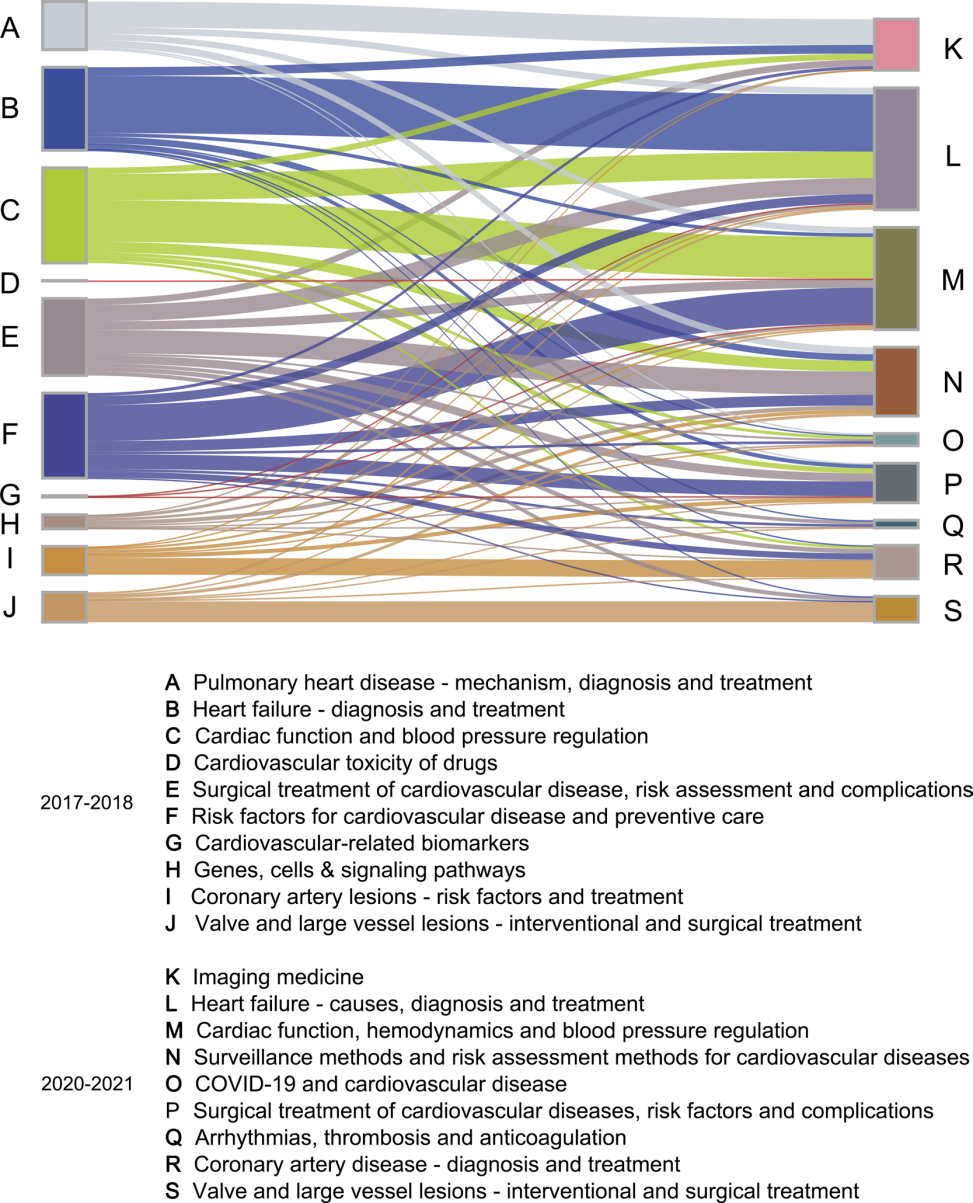
The citation relationships for identifying research overlap between two document clusters from 2017 to 2018 and 2020–2021 are presented through Sankey diagrams.COVID-19, Coronavirus disease 2019.

Figure 4 illustrates the crossover relationships among document clusters for 2017–2018 and 2020–2021. It juxtaposes the citations of documents published between 2020 and 2021 with those of documents published between 2017 and 2018. This figure demonstrates the presence of domain crossover, with each cluster displaying citation relationships with no fewer than three clusters from the opposing group. For instance, in 2017–2018, the cluster ‘*Surgical treatment of CVD, risk factors, and complications*’ cited literature from nine cluster categories, encompassing a broad spectrum of clinical, basic and population studies, of the largest number of citations came from cluster ‘*Surveillance methods and risk assessment methods for CVD*’.

## Discussion

This study offers a comprehensive analysis of cardiovascular research trends from 1944 to 2021. The rising global burden of CVD has driven significant advances, highlighting the need for continued scientific innovation. Using NLP, we identified key topics, clusters and their intersections across a large body of literature. By extending the timeline beyond previous studies, our findings provide deeper insights into the field’s evolution.

We observed a notable shift in focus, with clinical research increasingly emphasising early detection, prevention and minimally invasive treatments—reflecting a broader move towards translational and patient-centred approaches. Although basic research has grown more slowly, areas like haemodynamics, regenerative medicine and translational science have gained traction. The intersection of CVD and COVID-19 research illustrates the field’s responsiveness to emerging health challenges, driven by evolving needs, technology and societal support. While our findings support a genuine expansion of clinical cardiovascular research and a movement toward translational objectives, this trend is likely multifactorial, shaped by both internal scientific developments and external systemic factors.

COVID-19 has reshaped cardiovascular research priorities, prompting greater focus on disease interplay. The growth of imaging medicine highlights the role of modern diagnostics in advancing care. Interdisciplinary overlaps reveal the integration of diverse fields, fostering innovation and collaboration. In surgical research, focus has expanded to include risk assessment, monitoring and foundational science. Overall, our study underscores the need for ongoing reassessment of research themes. As science and health landscapes evolve, researchers must remain alert to emerging trends to stay at the forefront of the field.

### Dynamics and interdisciplinary progress in cardiovascular topics

Minimally invasive interventions for valvular disease represent the most rapidly advancing topic in clinical research, driven by continual technological and device innovations.^10 11^ These developments have improved patient outcomes by reducing postoperative complications and shortening recovery times, aligning with the goals of patient-centred care. Concurrently, imaging medicine has emerged as a key research cluster, significantly advancing in recent years. Enhanced imaging precision supports the development of minimally invasive procedures by enabling earlier detection of valvular pathologies, thus facilitating preventive strategies and personalised treatment planning.^14^

From 2011 to 2021, research on health monitoring has surged markedly, reflecting a broader shift toward early detection and disease prevention.^15^ This trend is supported by parallel advances in materials science,^16^ artificial intelligence^17^ and big data.^18^ Integrated health monitoring systems leveraging these technologies now empower patients to participate actively in chronic disease management, reducing hospitalisation rates and improving quality of life in CVD populations.

In basic research, haemodynamics, immunology and regenerative medicine have gained prominence over the past decade, reflecting the pursuit of novel therapeutic strategies. Innovations in regenerative medicine—such as bioengineered vascular grafts—support targeted tissue repair in early-stage CVD, contributing to disease prevention by slowing progression.^19^ These themes are prevalent in clinical and population studies.^20^ For example, risk factor research remains a consistent focus across topic and document cluster analyses, bridging basic, clinical and population research domains.

The integration of imaging, polygenic risk scores and multiomic strategies provides a high-dimensional, precision-driven and personalised approach to CVD risk assessment, exemplifying the rapid progression of translational medicine.^21^ Particularly amid the COVID-19 pandemic, translational medicine research has made notable strides in comprehending the intricate interplay between COVID-19 and CVD.^22^ Nearly all preceding document clusters have a citation relationship (figure 4), particularly in relation to conditions such as multisystem inflammatory syndrome.^13^

These interdisciplinary advances have significantly enriched cardiovascular science.^23^ By integrating knowledge and technologies from diverse fields, researchers can gain a more comprehensive understanding of the pathogenesis of CVDs and subsequently develop more effective, personalised treatment approaches. For instance, immunology-driven discoveries in inflammatory biomarkers now inform patient-centred protocols, enabling tailored anti-inflammatory therapies to reduce recurrent ischaemic events.^24^ Such collaboration across disciplines continues to propel meaningful scientific progress.

### Revolution in traditional cardiovascular topics

One of the main findings of this study is the steady growth of clinical research, showing a stronger focus on turning scientific discoveries into practical care. Core areas, such as coronary artery disease, heart failure, hypertension, arrhythmias and stroke, remain central in cardiovascular research, highlighting their ongoing importance. In particular, research on evidence-based care guidelines and treatment outcomes has expanded rapidly. Many clinical trials on new drugs, devices and technologies have led to better CVD outcomes.^3^

Turning research into practice is vital for improving patient care and health outcomes. To support this, health policies should create easier approval pathways and offer financial support for clinical trials of innovative therapies, as recommended by the WHO.^25^ Healthcare systems also need faster ways to apply new evidence in practice, following guidance from the European Society of Cardiology.^26^

In population studies, continued attention to hypertension and the rising interest in metabolic syndrome point to the need for a more complete approach to heart health. Today’s research goes beyond individual risk factors, using evidence-based strategies, clinical guidelines and insights from different disciplines. This broader approach has shifted attention to long-term issues such as disease trends, healthcare costs and quality of care.^27 28^

To fully benefit from interdisciplinary research, policies should fund projects that connect medicine, engineering, data science and public health. This could involve joint grants, team-based research requirements and partnerships between public and private sectors to speed up the use of new CVD treatments and technologies.^29^ In the long run, tackling preventable risk factors will need close coordination between clinical care and public health efforts.^28^

### Drivers of change in cardiovascular research topics

The growth of cardiovascular research is shaped by various factors, including medical demand, technological progress, funding and policy direction. A notable global trend is the increased allocation of resources to clinical research, often at the expense of basic and population studies,^3^ aiming to accelerate the translation of findings into healthcare policy and practice. The slower growth of basic research may partly reflect a corresponding decline in funding.^3^ As medical technologies advance and clinical demands rise, resources are increasingly directed towards research with immediate, tangible benefits—reflecting society’s urgent need for improved care and rapid application of scientific discoveries.

In the USA, despite CVD’s high burden of mortality and disability, research funding for CVD lags behind that for cancer, immunology and rare diseases. This disparity may stem from funders’ reluctance to support high-risk, innovative projects,^30^ despite the strong cost-effectiveness of cardiovascular research. Additionally, deficiencies in diversity, equity and inclusion may undermine the future of the field. Fostering a diverse, collaborative workforce is essential for addressing the global CVD burden and aligns with the interdisciplinary evolution of cardiovascular research hotspots.^31^

The COVID-19 pandemic exemplifies how medical crises can rapidly redirect research priorities. Funding and attention surged toward COVID-19–related CVD research, temporarily eclipsing other areas. The pandemic disrupted routine care and clinical trials but also prompted greater collaboration and adaptability within the research community.^32^ These developments highlight the need for more agile funding systems that track emerging trends, support innovation and enable timely responses to urgent health challenges. Ensuring sufficient resources for high-quality, exploratory research remains critical to advancing cardiovascular science.

### Limitations

This study provides a comprehensive overview, but several limitations should be noted. First, our analysis relied on publications indexed in the MEDLINE database via WoS, potentially omitting research not included in this source. However, the inclusion of over 2.5 million records lends strong support to our findings. Second, while expert input may introduce bias, we minimised this risk through the use of diverse expert panels and established methodologies.^3^ Third, the study focused exclusively on published literature, potentially overlooking ongoing or unpublished work. As a result, emerging topics with limited current output may be underrepresented. Additionally, the analysis is subject to inherent temporal delays and human resource constraints.^3^ Future studies could leverage real-time data and advanced analytical tools to address this. Fourth, our interdisciplinary analysis was limited to citation data from two discrete periods (2017–2018 and 2020–2021), selected to avoid disruptions caused by the COVID-19 pandemic and delayed post-2022 publications. However, this narrow timeframe may not adequately capture long-term interdisciplinary dynamics, which warrants broader temporal analysis in future research. Fifth, we did not examine other forms of interdisciplinary collaboration—such as co-authorship networks, methodological convergence or conceptual integration—nor did we incorporate alternative indicators to assess such interactions. Sixth, the study emphasised quantitative metrics (eg, publication counts) without assessing research quality or impact. Due to the limitations of bibliometric data, it was not feasible to disentangle the influence of funding policies or editorial decisions. Lastly, limiting the analysis to English-language publications may have excluded valuable insights from non-English-speaking regions. Future research should incorporate multilingual data to provide a more inclusive and globally representative picture of cardiovascular research.

## Conclusion

Our analysis provides a panoramic overview of the trends and evolution in cardiovascular research topics from 1944 to 2021. This reveals the key areas of focus and potential avenues for future research. Noteworthy trends include an increasing emphasis on early disease detection and prevention, exploration of minimally invasive treatments and the assessment of risk factors. The research landscape demonstrates signs of interdisciplinarity and integration as reflected in citation relationships. Given the evolving global burden of CVD, continuous research and innovation are imperative, with interdisciplinary collaboration playing a pivotal role in propelling scientific advancement.

## Supplementary material

10.1136/heartjnl-2025-325877 online supplemental file 1

## Data Availability

Data are available in a public, open access repository.

## References

[1] Vos T, Lim SS, Abbafati C (2020). Global burden of 369 diseases and injuries in 204 countries and territories, 1990–2019: a systematic analysis for the Global Burden of Disease Study 2019. Lancet.

[2] Roth GA, Mensah GA, Johnson CO (2020). Global Burden of Cardiovascular Diseases and Risk Factors, 1990-2019: Update From the GBD 2019 Study. J Am Coll Cardiol.

[3] Gal D, Thijs B, Glänzel W (2019). Hot topics and trends in cardiovascular research. Eur Heart J.

[4] Synnestvedt MB, Chen C, Holmes JH (2005). CiteSpace II: visualization and knowledge discovery in bibliographic databases. *AMIA Annu Symp Proc*.

[5] van Eck NJ, Waltman L (2010). Software survey: VOSviewer, a computer program for bibliometric mapping. Scientometrics.

[6] Gates AJ, Ke Q, Varol O (2019). Nature’s reach: narrow work has broad impact. Nature New Biol.

[7] Kozlowski D, Semeshenko V, Molinari A (2021). Latent Dirichlet allocation model for world trade analysis. PLoS One.

[8] Chen ZL (2022). Research and Application of Clustering Algorithm for Text Big Data. Comput Intell Neurosci.

[9] Shih H, Rajendran S (2019). Comparison of Time Series Methods and Machine Learning Algorithms for Forecasting Taiwan Blood Services Foundation’s Blood Supply. J Healthc Eng.

[10] Vahanian A, Beyersdorf F, Praz F (2022). 2021 ESC/EACTS Guidelines for the management of valvular heart disease. Eur Heart J.

[11] Otto CM, Nishimura RA, Bonow RO (2021). 2020 ACC/AHA Guideline for the Management of Patients With Valvular Heart Disease: Executive Summary: A Report of the American College of Cardiology/American Heart Association Joint Committee on Clinical Practice Guidelines. Circulation.

[12] Poulter NR, Prabhakaran D, Caulfield M (2015). Hypertension. Lancet.

[13] Nalbandian A, Sehgal K, Gupta A (2021). Post-acute COVID-19 syndrome. Nat Med.

[14] Donal E, Unger P, Coisne A (2025). The role of multi-modality imaging in multiple valvular heart diseases: a clinical consensus statement of the European Association of Cardiovascular Imaging of the European Society of Cardiology. Eur Heart J Cardiovasc Imaging.

[15] Visseren FLJ, Mach F, Smulders YM (2021). 2021 ESC Guidelines on cardiovascular disease prevention in clinical practice. Eur Heart J.

[16] Choi YS, Yin RT, Pfenniger A (2021). Fully implantable and bioresorbable cardiac pacemakers without leads or batteries. Nat Biotechnol.

[17] Yu KH, Beam AL, Kohane IS (2018). Artificial intelligence in healthcare. Nat Biomed Eng.

[18] Schüssler-Fiorenza Rose SM, Contrepois K, Moneghetti KJ (2019). A longitudinal big data approach for precision health. Nat Med.

[19] Hoang VT, Nguyen QT, Phan TTK (2025). Tissue Engineering and Regenerative Medicine: Perspectives and Challenges. MedComm.

[20] Zhang J, Bolli R, Garry DJ (2021). Basic and Translational Research in Cardiac Repair and Regeneration: JACC State-of-the-Art Review. J Am Coll Cardiol.

[21] Verma KP, Inouye M, Meikle PJ (2022). New Cardiovascular Risk Assessment Techniques for Primary Prevention: JACC Review Topic of the Week. J Am Coll Cardiol.

[22] Guo T, Fan Y, Chen M (2020). Cardiovascular Implications of Fatal Outcomes of Patients With Coronavirus Disease 2019 (COVID-19). JAMA Cardiol.

[23] Wan KK, Davis D, Lee TN (2022). A call to action for translational sciences in COVID-19 and future pandemics. Nat Rev Drug Discov.

[24] Ridker PM, Everett BM, Thuren T (2017). Antiinflammatory Therapy with Canakinumab for Atherosclerotic Disease. N Engl J Med.

[25] World Health Organization (2019). WHO guideline: recommendations on digital interventions for health system strengthening.

[26] Piepoli MF, Hoes AW, Agewall S (2016). 2016 European Guidelines on cardiovascular disease prevention in clinical practice: The Sixth Joint Task Force of the European Society of Cardiology and Other Societies on Cardiovascular Disease Prevention in Clinical Practice (constituted by representatives of 10 societies and by invited experts)Developed with the special contribution of the European Association for Cardiovascular Prevention & Rehabilitation (EACPR). Eur Heart J.

[27] Touyz RM, Schiffrin EL (2021). A Compendium on Hypertension: New Advances and Future Impact. Circ Res.

[28] Lemieux I, Després JP (2020). Metabolic Syndrome: Past, Present and Future. Nutrients.

[29] Mabry PL, Olster DH, Morgan GD (2008). Interdisciplinarity and systems science to improve population health: a view from the NIH Office of Behavioral and Social Sciences Research. Am J Prev Med.

[30] Nicholls M (2018). Funding of cardiovascular research in the USA: Robert Califf and Peter Libby - speak about cardiovascular research funding in the United States and what the latest trends are with Mark Nicholls. Eur Heart J.

[31] Chapman N, Thomas EE, Tan JTM (2022). A roadmap of strategies to support cardiovascular researchers: from policy to practice. Nat Rev Cardiol.

[32] Park JJH, Mogg R, Smith GE (2021). How COVID-19 has fundamentally changed clinical research in global health. Lancet Glob Health.

