# Supplementary material for: Analysis and prediction of cardiovascular research hotspots, trends and interdisciplinarity
Source: Heart. 2025 Aug 28;112(7):e325877. doi: 10.1136/heartjnl-2025-325877 (PMC13018832; doi:10.1136/heartjnl-2025-325877)
Supplement: online supplemental file 1 [file heartjnl-112-7-s001.docx]

**SUPPLEMENTAL MATERIALS**

***Analysis and Prediction of Cardiovascular Research Hotspots, Trends, and Interdisciplinarity***

Zeye Liu, Ziping Li, Hong Jiang, Guangyu Pan, Wenchao Li, Fengwen Zhang, Wenbin Ouyang, Shouzheng Wang, Cheng Wang, Xuanqi An, Anlin Dai, Ruibing Xia, Yakun Li, Xiaochun Sun, Yi Shi, Chengliang Yin, Xiangbin Pan

**List of Content**

1. **Supplementary Methods**
2. **Supplemental Figures**

- **Figures S1.** Schematic diagram of the main flow of the study.
- **Figures S2.** Topics with large growth for 2011–2021 and projections until 2027.

1. **Supplemental Tables**

- **Table S1.** Search terms and the number of corresponding literatures.
- **Table S2.** List of the 108 LDA topics.
- **Table S3.** The list of keywords from 1944 to 2021.
- **Table S4.** Glossary of Key Terms and Abbreviations.

**Supplementary Methods**

In this study, we employed two distinct yet complementary approaches: the k-means clustering algorithm and Latent Dirichlet Allocation (LDA)^1,2^.

1. ***k-means clustering algorithm***

Text clustering serves as a preparatory step in natural language processing (NLP), significantly influencing the subsequent analysis and processing of textual data. It is a well-established notion that within a common topic, sentences exhibit a higher frequency of similar words and phrases. Consequently, text clustering endeavours to gather these similar lexical items, grouping sentences that address the same subject matter into coherent clusters. Among widely used clustering methods, the k-means algorithm remains popular due to its computational efficiency. However, it is known to have limitations, particularly its sensitivity to local optima, which may result in suboptimal clustering—especially when dealing with high-dimensional, sparse vectors common in unstructured or semi-structured text. These conditions can lead to the unintended grouping of semantically unrelated documents. To address this, we carefully selected the number of clusters (n_clusters) using a combination of the elbow method and silhouette score analysis to balance cohesion and separation.

For the k-means model, we selected the optimal number of clusters (K) using the elbow method. Specifically, we tested clustering results with K ranging from 5 to 40 and listed the SSE (Sum of Squared Errors). As showed, the SSE drops significantly up to K = 20, after which the decrease becomes more gradual. Based on this inflection point, we chose K = 20 as the optimal setting for our study.

| **K** | **SSE** |
| --- | --- |
| 5 | 12470782 |
| 10 | 10127343 |
| 15 | 8925181 |
| **20** | **7816377** |
| 25 | 7566740 |
| 30 | 7394787 |
| 35 | 7356295 |
| 40 | 7336740 |

Key hyper-parameters of K-means are shown in the following table:

| **Parameter** | **Value** | **Description** |
| --- | --- | --- |
| n_clusters | 20 | Number of clusters and centroids |
| init | k-means++ | Initialization method |
| max_iter | 300 | Maximum iterations per run |
| tol | 0 | Convergence tolerance (Frobenius norm of centroid change) |

1. ***Latent Dirichlet Allocation (LDA)***

To ensure stable and accurate clustering outcomes, it is essential to identify appropriate initial cluster centers. In this context, we employed LDA, a probabilistic topic modeling technique, to complement traditional clustering methods. In our study, LDA was used to assign a unique topic distribution to each document and enrich these topics with relevant keywords, thereby enhancing the interpretability and thematic coherence of the clusters.

For the LDA model, we tested a range of topic numbers from 50 to 500. We evaluated each configuration based on its perplexity on a validation set and the average coherence across topics. The results show that when K = 300, the model achieved the lowest perplexity and the highest coherence score. This indicates that the topic distribution is both more accurate and more consistent at this setting. Therefore, we selected 300 topics as the optimal hyperparameter for our LDA model.

| **K** | **Perplexity** | **Coherence** |
| --- | --- | --- |
| 50 | 0.0016 | -4.0750 |
| 100 | 0.0018 | -4.1004 |
| 150 | 0.0016 | -4.0544 |
| 200 | 0.0016 | -4.0975 |
| 250 | 0.0016 | -4.0871 |
| **300** | **0.0015** | **-4.0316** |
| 350 | 0.0016 | -4.0362 |
| 400 | 0.0016 | -4.0683 |
| 450 | 0.0016 | -4.0689 |
| 500 | 0.0017 | -4.0755 |

Key hyper-parameters of LDA are shown in the following table:

| **Parameter** | **Value** | **Description** |
| --- | --- | --- |
| num_topics | 300 | Number of latent topics |
| passes | 2 | Corpus passes during training |
| chunksize | 500 | Documents per training batch |
| workers | 500 | Parallel processes (if None, all available cores) |
| iterations | 50 | Max iterations during inference |
| decay | 0.5 | Weight decay factor |
| offset | 1 | Controls early iteration learning rate |

1. ***Data Sources***

The dataset encompassed title, abstract, and keyword information for 2,512,445 cardiovascular publications spanning from 1944 to 2021, along with citation data for literature published from 2011 to 2021. This dataset was derived from a search using 40 cardiovascular terms based on Medical Subject Headings (MeSH) phrases provided by PubMed (**Table S1**). The data was sourced from the MEDLINE dataset of the Web of Science (WoS) Core Collection, under the data license held by the Chinese Academy of Medical Sciences & Peking Union Medical College.

1. ***Text Pre-processing***

Out of the initial 6,468,884 documents, we removed literature that lacked abstracts and eliminated duplicates using Digital Object Identifier (DOI) numbers and titles. This left 2,512,445 documents. We processed all titles and abstracts from these cardiovascular publications and extracted time information and noun phrases using a natural language-processing framework implemented in Python v3.8 software, utilized libraries such as nltk, jieba, and pandas for data preprocessing and analysis. During the modelling phase, we employed scikit-learn to construct the K-means clustering model and Gensim (Software Framework for Topic Modelling with Large Corpora) to build the LDA topic model. they are among the most popular libraries for machine learning and NLP applications (PPPNE and predicting multiple conformations via sequence clustering and AlphaFold2). A visual representation of the data analysis is shown in **Figure S1**.

In terms of temporal selection, our aspiration lies in having a sufficiently long-time span to gather and analyse cardiovascular-related publications. This approach ensures two primary objectives: first, a sufficient number of publications can be amassed for the study; second, the impact of short-term fluctuations or contingent factors on data interpretation can be mitigated, thereby enhancing the representativeness and reliability of the research. After exploring various combinations of years and their corresponding publication volumes, we settled on a span from 1944 to 2021. The year 1944 marked the end of World War II, when large-scale global turmoil subsided, allowing medical research resources to be redistributed and concentrated. Consequently, more physicians and scientists have been able to devote their energy to medical research. Subsequently, significant breakthroughs in the treatment and management of cardiovascular diseases gradually emerged, such as the introduction of Coronary Care Units (CCUs) in 1960, the inception of thrombolytic therapy in 1970, and the advent of percutaneous coronary intervention (PCI) in 1980. By setting the inclusion year to precede these milestones, we aimed to encapsulate the pivotal advancements in cardiovascular medicine while maintaining the objectivity and stability of our research.

In this study, no inclusion/exclusion criteria were applied specifically to the sources of publications; rather, a comprehensive approach was adopted to include content published across all journals. Given the substantial time span considered, various objective factors, such as each journal's predominant areas of interest, peer-review standards, and impact factors, have undergone significant changes. Additionally, the quantity and quality of scientific output from each journal fluctuated continuously during this period. Thus, arbitrarily excluding certain journals from the analysis could potentially introduce a substantial bias. Furthermore, a ‘hot topic’ refers to an area of research that attracts a significant number of researchers over a particular period, leading to a proliferation of studies and, consequently, a substantial volume of research articles. These articles are widely disseminated across various journals regardless of their impact factors. Consequently, we elected not to exclude any articles from our analysis, believing that this approach would provide a more comprehensive reflection of the evolution of research hotspots in the cardiovascular field.

1. ***Modelling analysis and prediction***

First, we applied the LDA approach^1,4^ to the textual content of all the titles and abstracts. This allowed us to group text fragments, identify topics, and associate documents. To enhance the accuracy and highlight category-specific features, we filtered out generic terms commonly used in most documents. This yielded highly specific text fragments that formed the basis of the identified topics.

The LDA model was implemented using the gensim.models.LdaMulticore library in Python. The keywords field (tokenized into individual words) was used to generate one-hot vector representations, which were then used to train the LDA model. We specified the number of topics as 300, with model parameters set as follows: chunksize = 500 and passes = 5.

Five cardiovascular experts (all of whom are listed as authors of this paper) then validated these topics based on the top 30 text fragments representing each topic (confirming the name of a topic only if consensus was reached among all experts). We have explicitly defined the criteria used for selecting the cardiovascular experts involved. These criteria included expertise in cardiovascular research, a minimum number of years of experience in the field, and a proven track record of publications in high-impact journals.

The experts identified topics and document clusters carefully through a series of steps. Initially, a preliminary set of topics was generated using the LDA model. This model allowed us to uncover latent topics within our corpus of cardiovascular research documents. Subsequently, the experts were presented with these topics and asked to refine them based on their domain knowledge and expertise. They were also asked to group related documents into clusters, using the identified topics as a guide. This iterative process involved multiple rounds of discussion and consensus-building, similar to the Delphi method, though more interactive.

To ensure the validity and reliability of the experts' judgments, we compared their clusters with those generated by the LDA model alone and found a high degree of agreement. We conducted a cross-review to merge similar topics. Subsequently, we used probabilistic analysis in the LDA approach to determine the number of documents contributing to each topic.

To predict future trends, we employed the AutoReg algorithm^5^.

Trend prediction was implemented using from statsmodels.tsa.ar_model import AutoReg with the parameter lags = 2. Due to missing year-specific data—especially in older publications lacking keywords or summaries—we assumed that the missing rate per year is constant and does not vary by LDA or K-means category. We calculated the total number of documents for each year, denoted as $c_{y}$​, using the entire dataset (including those with missing fields). We then counted the number of documents with complete summary fields, $c_{y}^{'}$, for each year $y$, and computed a weight $wy=\frac{c_{y}}{c_{y}^{'}}$ This weight was used to adjust the number of documents in each LDA or K-means category for each year to estimate true counts.

We then used the adjusted data to predict trends in three major research areas—clinical, basic, and population studies—over the next five years. Additionally, we generated word clouds based on keyword frequency across three periods (pre-2010, 2010–2020, and post-2020) to identify research hotspots.

Second, we scrutinised cardiovascular publications for 2017–2018 and 2020–2021. In each period, we calculated document similarity and grouped them based on text fragments in the titles and abstracts, utilising an adaptive cosine calculation and a hybrid document clustering algorithm^2,4^.

Five additional cardiovascular experts (not involved in identifying LDA topics, but all of whom are listed as the authors of this paper) named the clusters based on the top 30 text fragments representing each cluster (document clusters were confirmed only upon unanimous agreement among all experts). The results were visualised using a ring diagram. For each document cluster, we identified the most representative topic, aligned with the topics generated using the LDA approach.

To discern trends in more specialised research areas, we calculated the inverse document frequency (IDF) of the keywords by year. This technique aided in identifying research directions that, due to the limited number of studies, were challenging to discern through previous analyses^6^.

All experiments were conducted on a machine equipped with an Intel Core i7-10700, 8 cores, and 32 GB RAM. Python v3.8 was used to perform all operations in a local high-performance computing environment without an extranet connection.

1. ***Cross-relationship analysis***

We utilised citation relationships within the literature to ascertain the overlap of studies between the two document clusters identified using the hybrid document clustering algorithm. We separately calculated the number of papers in each cluster and determined the number of papers from 2017 to 2018 cited by papers from 2020 to 2021 for each cluster. These results are then presented in the form of a Sankey diagram^7^.

In our analysis, interdisciplinarity primarily refers to the integration and interaction of knowledge across distinct domains, including both within-medicine collaboration (e.g., cardiology, immunology, and neurology) and cross-domain integration involving fields such as biomedical engineering, data science, imaging technology, artificial intelligence, and public health. Specifically, we emphasize that:

Our citation-based analysis captures interdisciplinary linkages at the document level, where clusters of research topics show citation relationships suggestive of conceptual or methodological exchange across domains.

Examples include the convergence of clinical cardiovascular research with advances in imaging technologies, machine learning applications in diagnostics, bioengineering of vascular grafts, and multi-omics approaches to risk prediction.

However, we acknowledge that our method does not directly assess collaborative interdisciplinarity (e.g., co-authorship across departments) or methodological interdisciplinarity (e.g., combined use of clinical trial design with AI modelling).

1. ***Abbreviations***

All glossary of key terms and abbreviations are shown in **Table S4**.

**Reference**

1. Kozlowski D, Semeshenko V, Molinari A. Latent Dirichlet allocation model for world trade analysis. *PLoS One*. 2021;16:e0245393. doi: 10.1371/journal.pone.0245393

2. Chen ZL. Research and Application of Clustering Algorithm for Text Big Data. *Comput Intell Neurosci*. 2022;2022:7042778. doi: 10.1155/2022/7042778

3. Zhang S, Pyne S, Pietrzak S, Halberg S, McCalla SG, Siahpirani AF, Sridharan R, Roy S. Inference of cell type-specific gene regulatory networks on cell lineages from single cell omic datasets. *Nat Commun*. 2023;14:3064. doi: 10.1038/s41467-023-38637-9

4. Gal D, Thijs B, Glänzel W, Sipido KR. Hot topics and trends in cardiovascular research. *Eur Heart J*. 2019;40:2363-2374. doi: 10.1093/eurheartj/ehz282

5. Shih H, Rajendran S. Comparison of Time Series Methods and Machine Learning Algorithms for Forecasting Taiwan Blood Services Foundation's Blood Supply. *J Healthc Eng*. 2019;2019:6123745. doi: 10.1155/2019/6123745

6. Naeem MZ, Rustam F, Mehmood A, Mui Zzud D, Ashraf I, Choi GS. Classification of movie reviews using term frequency-inverse document frequency and optimized machine learning algorithms. *PeerJ Comput Sci*. 2022;8:e914. doi: 10.7717/peerj-cs.914

7. Gates AJ, Ke Q, Varol O, Barabási AL. Nature's reach: narrow work has broad impact. *Nature*. 2019;575:32-34. doi: 10.1038/d41586-019-03308-7

**
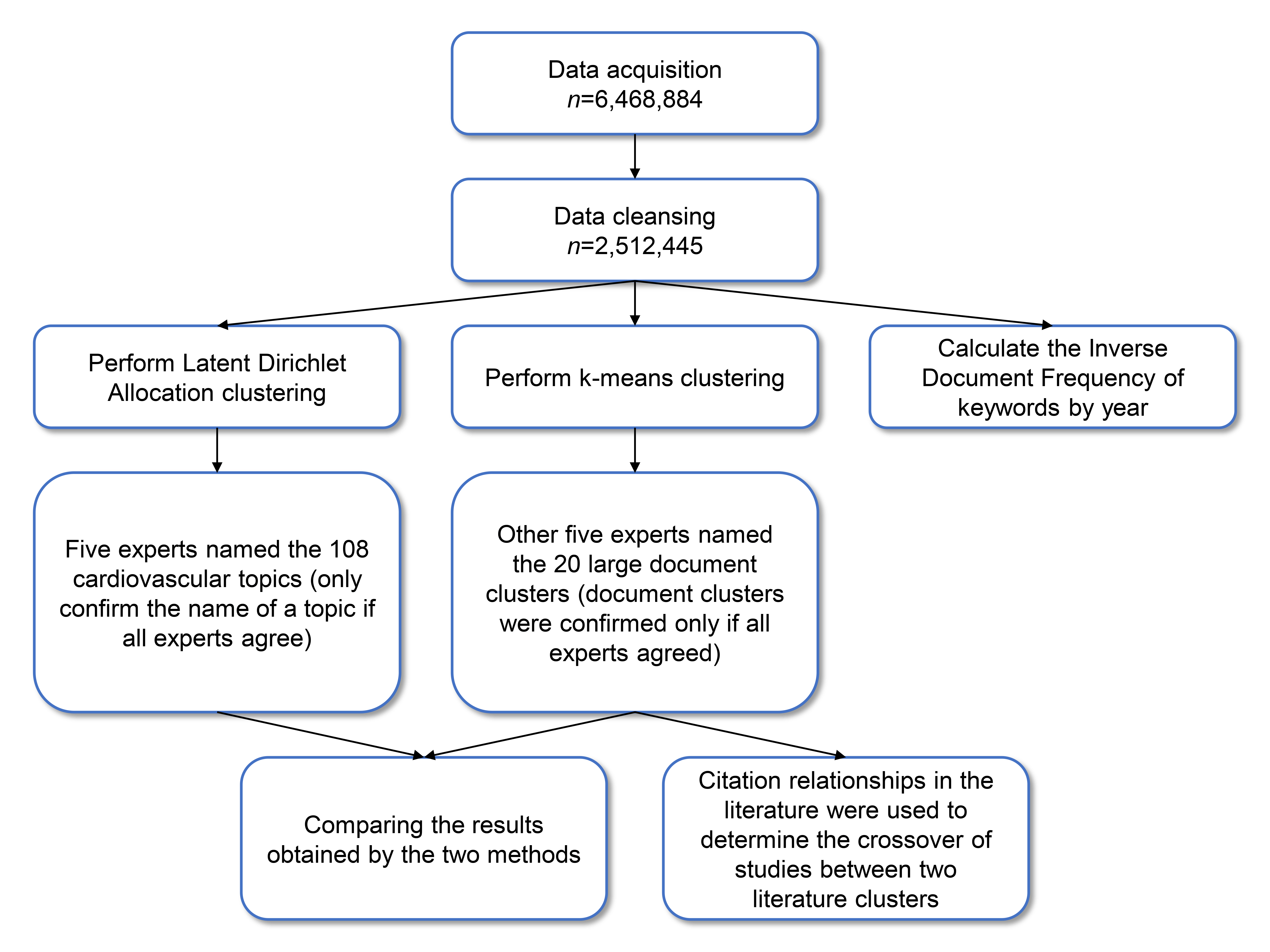
**

**Figures S1.** Schematic diagram of the main flow of the study.


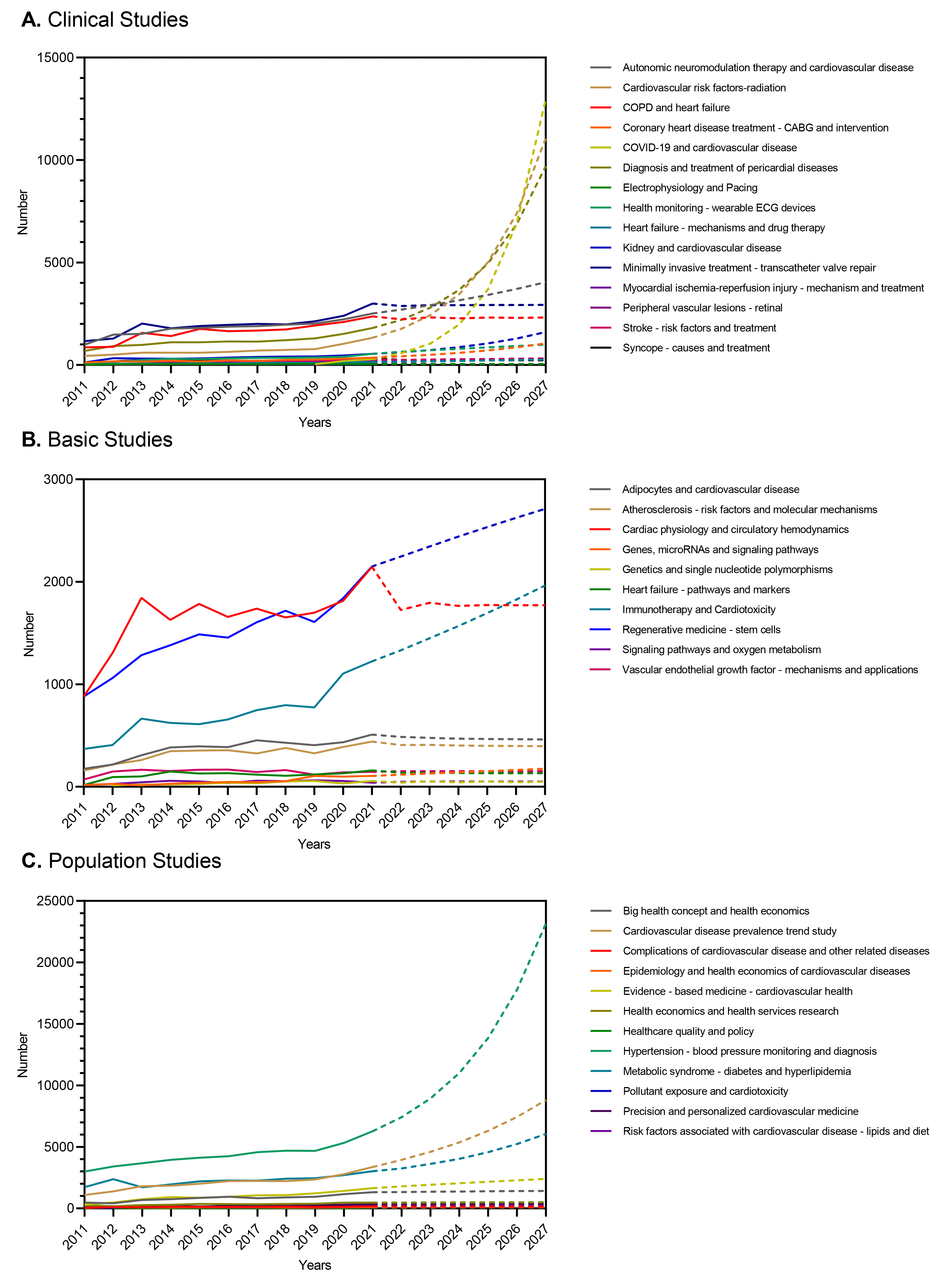


**Figures S2.** Topics with large growth for 2011–2021 and projections until 2027. The topics that increased more than two-fold in volume for 2011–2021 are shown. Solid lines represent actual values and dashed lines represent predicted values.

**Abbreviations:** CABG, coronary artery bypass graft; COPD, chronic obstructive pulmonary disease; COVID-19, Coronavirus disease 2019; ECG, electrocardiogram.

**Table S1.** Search terms and the number of corresponding literatures.

| Search terms | Number of documents |
| --- | --- |
| Arrhythmias, Cardiac | 109760 |
| Carcinoid Heart Disease | 907 |
| Cardiac Conduction System Disease | 7635 |
| Cardiac Disease | 307385 |
| Cardiac Disorder | 44162 |
| Cardiac Output, High | 19575 |
| Cardiac Output, Low | 22504 |
| Cardiac Tamponade | 10781 |
| Cardiomegaly | 27247 |
| Cardiomyopathies | 37642 |
| Cardiotoxicity | 14590 |
| Cardiovascular | 638360 |
| Cardiovascular Abnormalities | 27680 |
| Cardiovascular Diseases | 423602 |
| cardiovascular disorder | 62710 |
| Cardiovascular health | 115379 |
| Cardiovascular Infections | 23520 |
| Coronary Disease | 343973 |
| Endocarditis | 43155 |
| Heart | 1359327 |
| Heart Aneurysm | 22534 |
| Heart Arrest | 50522 |
| Heart Defects, Congenital | 70467 |
| Heart Diseases | 548302 |
| Heart Failure | 276569 |
| Heart Neoplasms | 47179 |
| Heart Valve Diseases | 63590 |
| Myocardial Infarction | 267978 |
| Myocardial Ischemia | 85500 |
| Myocardial Stunning | 3782 |
| Pericardial Effusion | 15777 |
| Pericarditis | 16564 |
| Pneumopericardium | 1227 |
| Pregnancy Complications, Cardiovascular | 21567 |
| Pulmonary Heart Disease | 62909 |
| Rheumatic Heart Disease | 17526 |
| Vascular | 834310 |
| Vascular Diseases | 339555 |
| Ventricular Dysfunction | 75440 |
| Ventricular Outflow Obstruction | 7692 |

There were 6,468,884 documents, and after removing literature with missing abstracts and removing duplicates using DOI numbers and titles, there were still 2,512,445 documents.

**Table S2.** List of the 108 LDA topics.

| Study Categories | LDA Topics |
| --- | --- |
| Clinical | Tetralogy of Fallot - diagnosis and treatment |
| Clinical | Diagnosis and treatment of pericardial diseases |
| Clinical | Cerebrovascular disease |
| Clinical | COPD and heart failure |
| Clinical | Coronary physiology - flow reserve fraction |
| Clinical | Minimally invasive treatment-TAVI |
| Clinical | Vascular malformations - diagnosis and treatment |
| Clinical | Acute coronary syndrome/myocardial infarction-risk factors and diagnosis |
| Clinical | Pulmonary vascular disease - thromboembolism and pulmonary hypertension |
| Clinical | Minimally invasive treatment - interventional treatment of valve diseases |
| Clinical | Organ transplantation - infection and immunity |
| Clinical | Cardiovascular risk factors - radiation |
| Clinical | Syncope - causes and treatment |
| Clinical | Imaging medicine - new approaches to cardiovascular ultrasound |
| Clinical | Trauma - vascular injury |
| Clinical | Imaging medicine - clinical applications of echocardiography |
| Clinical | Coronary artery malformation - diagnosis and treatment |
| Clinical | Autonomic neuromodulation therapy and cardiovascular disease |
| Clinical | Arrhythmia - ventricular arrhythmia diagnosis and treatment |
| Clinical | Cardiovascular and immune diseases - lupus erythematosus |
| Clinical | Biomarkers - diagnosis and prediction |
| Clinical | Acute coronary syndrome - OCT and interventional treatment |
| Clinical | Regenerative medicine - angiogenesis |
| Clinical | Kidney and cardiovascular disease |
| Clinical | Congenital cardiovascular malformations - diagnosis and Treatment |
| Clinical | Thrombosis and embolism - diagnosis and treatment |
| Clinical | Cardiotoxicity of chemotherapy drugs |
| Clinical | Aneurysm - causes and treatment |
| Clinical | Cardiovascular effects of anesthesia |
| Clinical | Surgical treatment of congenital heart disease and complications |
| Clinical | Heart block - causes and treatment |
| Clinical | Adjunctive therapy for cardiovascular diseases |
| Clinical | Cardiovascular disease during pregnancy and delivery |
| Clinical | Cardiovascular effects of anti-inflammatory drugs |
| Clinical | Health monitoring - wearable ECG devices |
| Clinical | Coronary heart disease treatment - CABG and intervention |
| Clinical | Bleeding and vascular complications |
| Clinical | Arrhythmia treatment - drugs and defibrillators |
| Clinical | Peripheral vascular lesions-retinal |
| Clinical | Heart transplantation - complication control |
| Clinical | Electrophysiology and pacing |
| Clinical | Stroke - risk factors and treatment |
| Clinical | Heart failure - mechanisms and drug therapy |
| Clinical | Cerebrovascular lesions-vascular dementia |
| Clinical | Hematological disease |
| Clinical | Minimally invasive treatment of valve disease |
| Clinical | Vascular calcification - mechanism and treatment |
| Clinical | Congenital cardiovascular malformations - diagnosis and treatment |
| Clinical | Imaging medicine - radiology & CT |
| Clinical | Malignant tumors of the cardiovascular system |
| Clinical | Imaging medicine - MRI & nuclear medicine |
| Clinical | Cardiovascular effects of other systemic diseases |
| Clinical | Cardiotoxicity of chemotherapy drugs |
| Clinical | Infectious diseases and the bacterial microenvironment |
| Clinical | Sleep breathing disorder syndrome |
| Clinical | Blood flow reconstruction and vascular stents |
| Clinical | Intelligent medical |
| Clinical | Valve diseases - surgical treatment and complications |
| Clinical | COVID-19 and cardiovascular disease |
| Clinical | Coronary interventional stenting - efficacy and complications |
| Clinical | Aging of the cardiovascular system - manifestations and mechanisms |
| Clinical | Cardiac arrhythmias-implantable defibrillator applications and complications |
| Clinical | Myocardial ischemia-reperfusion injury - mechanism and treatment |
| Clinical | Treatment of thromboembolism and vascular malformations |
| Clinical | Drug - related cardiovascular safety and tolerability |
| Clinical | Angina pectoris - diagnosis and treatment |
| Basic | IgG4 - related cardiovascular disease |
| Basic | Resolvin, Inflammation, and cardiovascular disease |
| Basic | Cardiac physiology and circulatory hemodynamics |
| Basic | Immunotherapy and cardiotoxicity |
| Basic | Vasoactive drugs - mechanistic studies |
| Basic | Histology and embryology - heart development |
| Basic | Anatomy - cardiovascular |
| Basic | Regenerative medicine - stem cells |
| Basic | Adipocytes and cardiovascular disease |
| Basic | Vascular endothelial cells and angiogenesis |
| Basic | Atherosclerosis - risk factors and molecular mechanisms |
| Basic | Signal transduction and cardiac proteins |
| Basic | Signaling pathways and oxygen metabolism |
| Basic | Metabolism of the cardiovascular system |
| Basic | Genes, microRNAs and signaling pathways |
| Basic | Signaling and ion channels |
| Basic | Vascular endothelial growth factor - mechanisms and applications |
| Basic | Atherosclerosis and occlusion - mechanisms and treatment |
| Basic | Genetics and single nucleotide polymorphisms |
| Basic | Heart failure - pathways and markers |
| Basic | Electrophysiology - ion channels and cell protection |
| Population | Metabolic diseases and cardiovascular health |
| Population | Cardiovascular disease prevalence trend study |
| Population | Hypertension - blood pressure monitoring and diagnosis |
| Population | Evidence - based medicine - cardiovascular health |
| Population | Burden of disease and epidemiology - cardiovascular disease |
| Population | Big health concept and health economics |
| Population | Metabolic syndrome - diabetes and hyperlipidemia |
| Population | Global health study - cardiovascular hazards of environmental pollution |
| Population | Precision and personalized cardiovascular medicine |
| Population | Health economics and health services research |
| Population | Pollutant exposure and cardiotoxicity |
| Population | Complications of cardiovascular disease and other related diseases |
| Population | Community health care and public health |
| Population | Factors associated with cardiovascular health - diet and exercise |
| Population | Healthcare quality and policy |
| Population | Risk factors associated with cardiovascular disease - lipids and diet |
| Population | Treatment and rehabilitation - cost benefit analysis |
| Population | Epidemiology and health economics of cardiovascular diseases |
| Population | Air pollution and cardiovascular health |
| Population | Cardiovascular-related nutrition - fatty acids |
| Population | The cardiovascular impact of mental illness and mental health |

**Abbreviations:** CABG, coronary artery bypass graft; COPD, chronic obstructive pulmonary disease; COVID-19, Coronavirus disease 2019; CT, computed tomography; ECG, electrocardiogram; LDA, latent Dirichlet allocation; MRI, magnetic resonance imaging; OCT, optical coherence tomography; TAVI, transcatheter aortic valve implantation.

**Table S3.** The list of keywords from 1944 to 2021.

| Year | Keywords 1 | Keywords 2 | Keywords 3 |
| --- | --- | --- | --- |
| 2021 | Multisystem inflammatory syndrome | Multisystem inflammatory syndrome in children | N6-methyladenosine |
| 2020 | 2019-nCoV | Coronavirus infections | Severe Acute Respiratory Syndrome |
| 2019 | Intracranial aneurysm | 4D flow CMR | Ablation index |
| 2018 | Cranial nerve | Unruptured intracranial aneurysm | Superior cerebellar artery |
| 2017 | Confidence intervals | Reflex syncope | Flammer syndrome |
| 2016 | Pathology section | Gerotarget | HR (Hazard Ratio) |
| 2015 | Fellowship training | Angiogenesis effect | Clinical competence |
| 2014 | Pediatric interventions | Angiogenesis effect | Myocardial ischemia and infarction (IHD) |
| 2013 | Left ventricle/ventricular | Fetal bovine serum | Phosphate-buffered saline |
| 2012 | Intensity-modulated radiation therapy | Medical imaging | Surgical procedures, minimally invasive |
| 2011 | General | Antiarrhythmic therapy | Coronary artery surgery |
| 2010 | Whole blood viscosity | Sonazoid | Pegaptanib |
| 2009 | Cardiac surgical procedures/methods | Acute disease | Tracheal intubation |
| 2008 | Equipment design | Combined modality therapy | Heart failure/therapy |
| 2007 | Study group | Vertebrobasilar insufficiency | Vascular patterning |
| 2006 | Investigators | Leukotriene D4 receptor | Coronary surgery |
| 2005 | Genetics and reproduction | Popular approach/source | Bioethics and professional ethics |
| 2004 | Non-programmatic | Bioethics and professional ethics | Empirical approach |
| 2003 | NASA discipline: Neuroscience | Investigators | Bioethics and professional ethics |
| 2002 | NASA discipline: Cell biology | Leukotriene D4 receptor | Non-programmatic |
| 2001 | NASA discipline: Neuroscience | NASA discipline: Neuroscience | NASA discipline: Cardiopulmonary |
| 2000 | Smoking - Women | Mental health therapies | NASA discipline: Musculoskeletal |
| 1999 | Contraceptive agents, progestin - Pharmacodynamics | Cardiovascular effects - Women | NASA discipline: Musculoskeletal |
| 1998 | Oral contraceptives, combined - Pharmacodynamics | Levonorgestrel - Pharmacodynamics | Contraceptive agents, female - Pharmacodynamics |
| 1997 | Gestodene | Oral contraceptives - Administration and dosage | NASA discipline: Regulatory physiology |
| 1996 | NASA program: Space biology | Desogestrel | Error sources |
| 1995 | Contraceptive implants | Mental health therapies | Adolescents, female |
| 1994 | NASA program: Space physiology and countermeasures | Contraceptive agents, female - Pharmacodynamics | Hormone antagonists |
| 1993 | Methodological studies | NASA discipline number 14-10 | Western Africa |
| 1992 | Methodological studies | Clinic activities | Contraceptive methods - Beneficial effects |
| 1991 | Oral contraceptives, combined - Pharmacodynamics | Method acceptability | Maternal-child health services |
| 1990 | Pregnancy, ectopic | Contraceptive methods - Pharmacodynamics | Methodological studies |
| 1989 | Length of life | Pelvic infections | Pregnancy, second trimester |
| 1988 | Population at risk | Bacterial and fungal diseases | Reproductive behavior |
| 1987 | Oral contraceptives - Complications | Macroeconomic factors | Genital effects, female |
| 1986 | Organic chemicals | Social behavior | Research and development |
| 1985 | Baby Fae | Genital effects, female | Contraceptive methods - Indications |
| 1984 | Reproductive control agents | Loma Linda University Medical Center | Popular approach/source |
| 1983 | Urogenital surgery | Religious approach | Abortifacient agents |
| 1982 | High-risk women | Reproductive control agents | Autoimmune response |
| 1981 | Summary report | Acceptor characteristics | Estrogens - Side effects |
| 1980 | Estrogens - Therapeutic use | Contraceptive mode of action | Acceptor characteristics |
| 1979 | Adolescents, female | Central nervous system effects | Dermatological effects |
| 1978 | Estrogens - Therapeutic use | Oral contraceptives - Indications | Curettage |
| 1977 | Renin-angiotensin-aldosterone effects | Estrogens - Side effects | Vaginal diaphragm |
| 1976 | Mestranol - Side effects | Estrogens - Therapeutic use | Cervix |
| 1975 | Prostaglandins - Administration and dosage | Prostaglandins - Administration and dosage | Thrombosis - Etiology |
| 1974 | Prostaglandins - Administration and dosage | Abortion, spontaneous | Amines |
| 1973 | Ovarian effects | Ethinyl estradiol - Side effects | Renin-angiotensin-aldosterone effects |
| 1972 | Mestranol - Side effects | Vaginal diaphragm | Nuptiality |
| 1971 | Contraceptive agents, estrogen - Side effects | Tubal occlusion | Pregnancy, first trimester |
| 1970 | Contraceptive mode of action | Tubal occlusion | Norethynodrel |
| 1969 | Melasma | Use-effectiveness | Diethylstilbestrol |
| 1968 | Norethynodrel | Cytologic effects | Mestranol - Side effects |
| 1967 | Ovarian effects | Mestranol - Side effects | Ovarian effects |
| 1966 | Monitoring systems | Dermatological effects | Obstetrical surgery |
| 1965 | Urography | Pronethalol | Myocardial diseases, primary |
| 1964 | Aortic valve diseases | Social conditions | Kinetocardiography |
| 1963 | Heart surgery/equipment and supplies | Ganglionic blockers | Brain injury, acute |
| 1962 | Vascular diseases, peripheral/radiography | Blood circulation - Pharmacology | Potassium/blood |
| 1961 | Norepinephrine/pharmacology | Vascular diseases, peripheral - Blood | Electrocardiography - Pharmacology |
| 1960 | Coronary vessels/effect of drugs on | Myocardium - Pharmacology | Cardiac patients - Rehabilitation |
| 1959 | Defects, congenital/surgery | Acetazolamide/related compounds | Lung diseases - Complications |
| 1958 | Endocarditis, bacterial/etiology and pathogenesis | Vessels - Effect of drugs on | Defects, congenital - Effect of drugs on |
| 1957 | Auricular fibrillation/etiology and pathogenesis | Body fluid balance | Lipoproteins - In blood |
| 1956 | Adrenal cortex/hormones | Cardiac enlargement - Complications | Autonomic drugs - Effects |
| 1955 | Vascular diseases peripheral/therapy | Cardiac enlargement - Complications | Procaine amide - Effects |
| 1954 | Mitral valve/stenosis | Vascular diseases peripheral | Blood circulation - Diseases |
| 1953 | Procaine/derivatives | Heart disease - Complications | Heart disease - Surgery |
| 1952 | Heart in various diseases | Resins | Heart disease - Prevention and control |
| 1951 | Dicumarol | Heart - Effect of drugs on | Heart disease, congenital |
| 1950 | Heart/effects of drugs on | Glycosides, cardiac | Heart valves and valvulotomy |
| 1949 | Cardiovascular system/diseases | Pregnancy - Heart in | Arrhythmia - Auricular flutter |
| 1948 | Coronary vessels/occlusion | Heart - Hypertrophy | Heart - Roentgenography |
| 1947 | Heart/disease | Thrombosis - Coronary | Heart - Abnormalities |
| 1946 | Heart/effects of drugs | Heart - Effects of drugs | Cardiovascular diseases - Syphilis |
| 1945 | Heart/diseases | Heart - Disease | Heart - Beat |
| 1944 | Multisystem inflammatory syndrome | Rheumatic fever - Cardiac complications | Cardiovascular system - Physiology |

Only the top three of the most frequent keywords are displayed.

**Table S4.** Glossary of Key Terms and Abbreviations.

| Glossary of Key Terms & Abbreviation | Full Form |
| --- | --- |
| CABG | Coronary Artery Bypass Grafting |
| CCUs | Coronary Care Units |
| COPD | Chronic Obstructive Pulmonary Disease |
| COVID-19 | Coronavirus Disease 2019 |
| CT | Computed Tomography |
| CVD | Cardiovascular Disease |
| DOI | Digital Object Identifier |
| ECG | Electrocardiogram |
| IDF | Inverse Document Frequency |
| LDA | Latent Dirichlet Allocation |
| MeSH | Medical Subject Headings |
| MRI | Magnetic Resonance Imaging |
| NLP | Natural language processing |
| OCT | Optical Coherence Tomography |
| PCI | Percutaneous Coronary Intervention |
| TAVI | Transcatheter Aortic Valve Implantation |
| WoS | Web of Science |
